# 大气颗粒物滤膜中极性有机物在线衍生装置的设计与应用

**DOI:** 10.3724/SP.J.1123.2021.03009

**Published:** 2022-01-08

**Authors:** Han ZHANG, Xu LIAO, Lai WEI, Zixing ZHANG, Hongyun REN, Xian ZHANG

**Affiliations:** 中国科学院城市环境研究所环境与健康重点实验室, 福建 厦门 361021; Key Laboratory of Urban Environment and Health, Institute of Urban Environment, Chinese Academy of Sciences, Xiamen 361021 China; 中国科学院城市环境研究所环境与健康重点实验室, 福建 厦门 361021; Key Laboratory of Urban Environment and Health, Institute of Urban Environment, Chinese Academy of Sciences, Xiamen 361021 China; 中国科学院城市环境研究所环境与健康重点实验室, 福建 厦门 361021; Key Laboratory of Urban Environment and Health, Institute of Urban Environment, Chinese Academy of Sciences, Xiamen 361021 China; 中国科学院城市环境研究所环境与健康重点实验室, 福建 厦门 361021; Key Laboratory of Urban Environment and Health, Institute of Urban Environment, Chinese Academy of Sciences, Xiamen 361021 China; 中国科学院城市环境研究所环境与健康重点实验室, 福建 厦门 361021; Key Laboratory of Urban Environment and Health, Institute of Urban Environment, Chinese Academy of Sciences, Xiamen 361021 China; 中国科学院城市环境研究所环境与健康重点实验室, 福建 厦门 361021; Key Laboratory of Urban Environment and Health, Institute of Urban Environment, Chinese Academy of Sciences, Xiamen 361021 China

**Keywords:** 气相色谱, 质谱, 硅烷化反应, 在线衍生, 极性有机物, 大气颗粒物, 装置, gas chromatography (GC), mass spectrometry (MS), silylation reaction, online derivation, polar organic compounds, atmospheric particulate matter, device

## Abstract

设计制作了一套用于气相色谱-质谱(GC-MS)分析极性有机物的在线衍生装置,并将其应用于大气颗粒物样品中极性有机物的检测。将大气颗粒物滤膜样品置于GC-MS进样口,通过使用套针组件,匀速引入气态衍生试剂*N*-甲基-*N*-(三甲基硅烷)三氟乙酰胺(MSTFA),使其在衬管内于310 ℃下与待测物接触,10 min即可完成硅烷化在线反应。反应过程中,色谱柱箱保持低温,衍生产物得以在柱头保留,反应完成后色谱柱箱程序升温,使衍生产物直接进行后续分离检测。应用在线衍生装置建立有机酸分析方法,获得了一元酸、二元酸、芳香酸、醇等极性有机物的检测信息,涵盖了大气化学分析常见的大部分目标化合物。该方法检出限为0.02~0.53 mg/L,线性相关系数为0.976~0.996,日内、日间RSD为0.27%~7.28%,适用于批量大气环境样品检测。与传统离线衍生技术相比,本装置使衍生反应处于高温惰性气体氛围,排除空气中水分对衍生试剂的损耗和衍生产物降解风险,反应稳定、效率高;固体滤膜上有机物进行热解吸的同时完成在线衍生,样品需求量小,操作简单,零有机试剂污染,并有望应用于醇类、酚类等多种极性有机化合物的分析。此外, 该装置搭建简单,可模块化设计,适用于不同品牌气相色谱仪,具有商业化推广前景。

## Abstract (en)

An online derivatization device for the analysis of polar organic compounds by gas chromatography-mass spectrometry (GC-MS) is designed. The derivatization reaction occurs in the hot GC injection port, and this is also known as injection port derivatization (IPD). IPD is usually performed in two ways: 1) direct IPD and 2) ion-pair extraction, followed by IPD. In both cases, the derivatization reagent reacts in liquid form. However, a method for online derivatization using gaseous derivatization reagents is provided. A special needle is designed and placed on the carrier gas transfer line to the injection port. The carrier gas is introduced into a glass bottle containing the derivative reagent (*N*-methyl-*N*-(trimethylsilyl)trifluoroacetamide, MSTFA), and then, the gaseous derivative reagent in the headspace is pressed out and introduced into the injection port of the GC instrument at a constant speed. The filter to be analyzed is placed directly in the liner, and the polar organic compounds on the filter react with gaseous MSTFA at 310 ℃ for 10 min in the injection port. During derivatization, the column oven is maintained at room temperature, and all the derivatives stay on the column head. When the reaction is complete, the MSTFA supply is ceased. The oven temperature is programmed, and the solvent delay is set until the excessive MSTFA is removed. The derivatives are allowed to pass through the column and analyzed by the MS detector. To prevent a large number of derivative reagents from entering the column, the injection port is set in split mode with a split ratio of 5:1. Variables such as the injection-port temperature and derivatization time are investigated. The GC-MS responses of the ten silylated derivatives increase with increasing injection-port temperature (290-310 ℃), indicating that high temperatures can enhance the silylation efficiency. The derivatization times were also investigated. The GC-MS responses increased with an increase in the reaction time from 0 to 10 min, while higher temperatures or longer reaction times lead to the loss of some derivatives. The reproducibility of the derivatization reaction was 0.27% to 7.28%, and the linear correlation coefficient was 0.976-0.996. This device can be used for the online silylation of most polar organic compounds such as organic acids, alcohols, and phenols. The advantage of this device over offline derivatization is that the derivatization reagent and derivatives are protected by helium, which eliminates the risk of decomposition caused by moisture in the air, and the high temperature assists the reaction. The analytes were directly desorbed on the filter and derived online, and the sample quantity required was only 1/200 of that in the traditional solvent extraction method. Meanwhile, only the gaseous part of the headspace in the derivative reagent bottle was used, and the amount of derivative reagent was greatly reduced. Additionally, the operation is simple and solvent free, the entire analytical procedure was executed in a “green” manner. A PM2.5 filter was analyzed, and 26 different polar compounds were successfully derived, including monoacids, binary acids, aromatic acids, and alcohols, covering most of the common target polar compounds in atmospheric chemical analysis. Polyols such as glucose and sorbose that bear 5-6 hydroxyl groups and have large steric hindrance were also successfully derived. This device is expected to be an efficient and convenient analytical tool for tracing the sources of organic matter in atmospheric particles such as soil dust, biomass combustion, cooking oil smoke, and automobile exhaust, or for investigating atmospheric photochemical reactions. This gas-phase derivatization provides new insights for the development of chromatographic analysis methods for polar compounds. This device is simple and modular, and it has a wide range of applications; it is suitable for different brands of gas chromatographs and has great prospects for commercialization.

有机物是大气颗粒物的重要组成部分。目前从分子水平上鉴别出来的有机物大致可分为15类,约占颗粒物质量的10%,包括烷烃、多环芳烃、霍烷类、脂肪醇、脂肪酸、芳香酸、二元羧酸、多元酸、醛酮类、糖类和持久性有机物等^[1]^。颗粒物中单种有机组分的含量极低,通常在0.1~100 ng/m^3[2]^,主要通过溶剂萃取、净化和浓缩后进行色谱分析,操作繁琐,样品需求量大^[3]^。与溶剂萃取相比,热解吸技术分析颗粒物中有机物具有一定的优越性:零有机溶剂污染,操作简单,样品量少,样品利用率接近100%。该技术最早由韩国科学家Ho等^[3,4]^提出,即将大气颗粒物滤膜样品直接放入气相色谱进样口,利用进样口的高温将滤膜上的有机物引入色谱分析;该技术被Cao等研究组广泛使用^[5,6,7]^。然而目前热解吸技术主要局限于非极性有机物分析,极性有机物一般需要离线衍生后方可利用气相色谱-质谱(GC-MS)分析^[8,9,10]^,所以热解吸技术较少应用于极性化合物的分析研究。有机酸是大气颗粒物中含量最丰富的一类极性化合物,也是二次有机气溶胶的重要组分,约占其颗粒物总质量的20%~60%。有机酸的挥发性低、极性强,在气粒分配中更容易沉积到细颗粒物中,因具有强吸湿性可改变颗粒物的粒径分布,其含量常用于评价气溶胶的老化程度,在大气颗粒物研究中占有重要地位^[11]^。

本研究设计制作了一套用于GC-MS分析极性有机物的在线衍生装置,在进样口热解吸技术基础上,引入气态衍生模式,实现大气颗粒物滤膜样品中有机酸、醇类、酚类等极性有机化合物的快速在线衍生,拓宽了热解吸技术的应用范围,为在线衍生色谱技术提供了新思路。

## 1 实验部分

### 1.1 仪器、试剂与材料

仪器:7890A-5975C气相色谱-质谱联用仪(安捷伦,美国),TH1000H大流量采样器(武汉天虹)。标准样品:丙二酸、戊二酸、己二酸、辛二酸、壬二酸、邻苯二甲酸、正十六酸、正十八酸(上海国药)、顺蒎酸、蒎酮酸(Dr. Ehrenstorfer公司,德国)。衍生试剂:*N*-甲基-*N*-(三甲基硅烷)三氟乙酰胺(MSTFA)(Accu Standard,美国)。材料:三通(1/16 in,世伟洛克,美国);SS-41GXS1四通阀门(世伟洛克,美国);不锈钢针(长100 mm,针尖上方侧开孔,孔直径2 mm)不锈钢管道(外径0.75 mm,内径0.5 mm);去活石英毛细管柱(内径0.32 μm);QMA石英滤膜(8 in×10 in,Whatman,英国)。

### 1.2 样品采集

将石英滤膜置于马弗炉中除去有机质(500 ℃, 4 h),使用大流量采样器连续采样24 h。采样完成后以铝膜密封,置于-20 ℃冰箱中待用,测试前冷冻干燥。测试时,以直径1.5 cm的打孔器取样后剪裁成条,使之适合衬管装载。所有条件实验以空白膜加标完成。

### 1.3 极性化合物在线衍生装置工作流程和条件

极性化合物在线衍生装置如图1所示。室温下,将滤膜置于进样口内衬管中,以载气吹扫。开启四通阀门3,利用进样口载气6将衍生试剂瓶5内顶空的气态衍生试剂吹入衬管。进样口温度由室温快速升高至310 ℃,保持10 min以完成在线衍生反应。为避免大量衍生试剂进入色谱柱,进样口采用分流模式。衍生过程中,色谱柱炉温保持35 ℃,使衍生产物在柱头保留。衍生完成后,切换阀门3,关闭衍生试剂,转换为纯氦气载流,启动程序升温。

**图 1:**
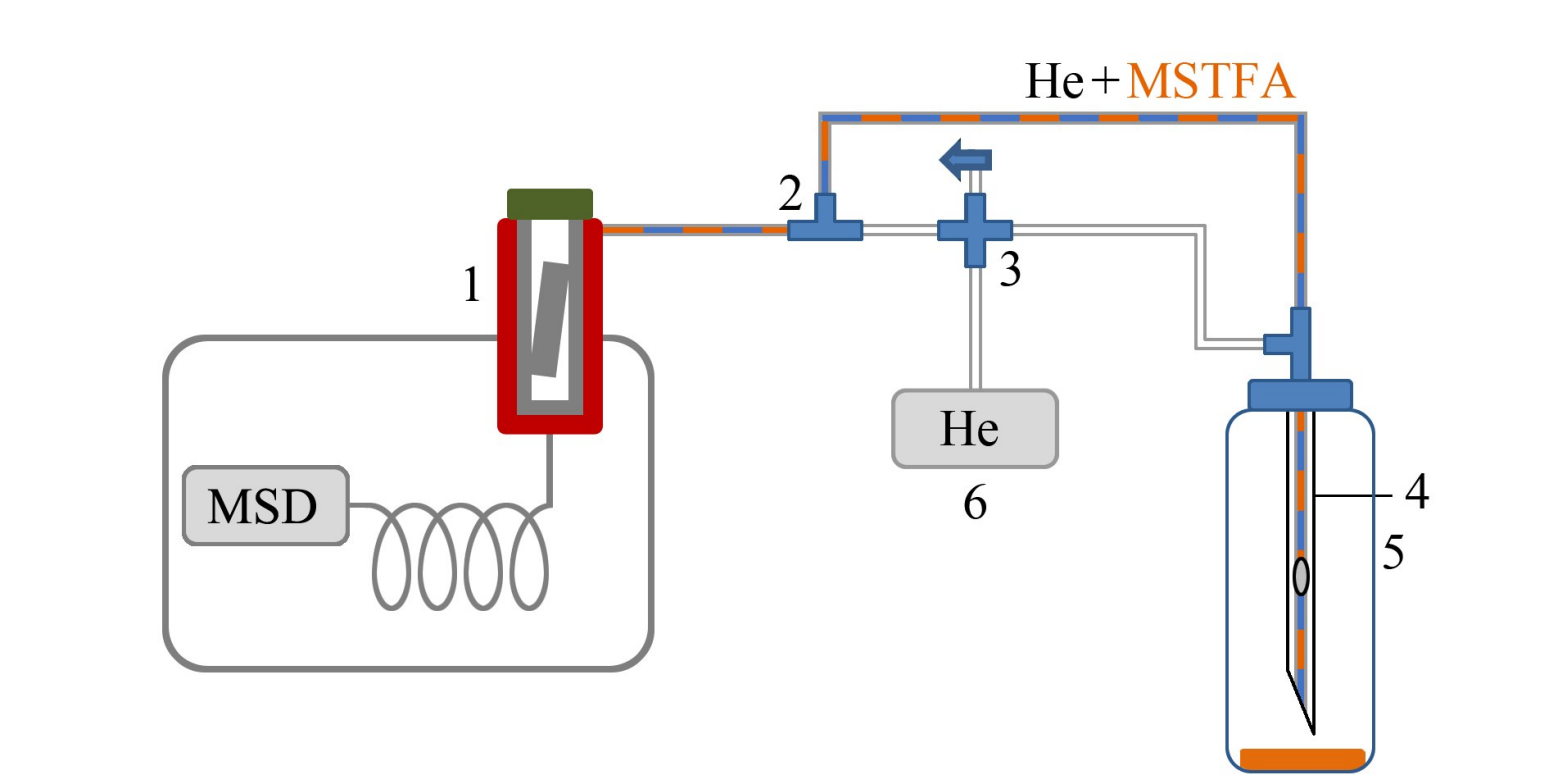
大气颗粒物滤膜中极性有机物在线分析装置图

气相色谱条件如下:色谱柱为DB-5MS(30 m×0.25 mm×0.25 μm),载气为氦气,进样口为分流模式,分流比5∶1,载气(载气+衍生试剂)流量为1 mL/min, GC柱温以35 ℃保持1 min,再以15 ℃/min升至280 ℃。

质谱条件:离子源温度230 ℃,四极杆温度150 ℃,接口温度280 ℃,质荷比(*m/z*)为30~500, 电子轰击电离(EI)源条件为70 eV,扫描模式为SCAN和SIM扫描,溶剂延迟6 min。

## 2 结果与讨论

### 2.1 衍生试剂选择

极性化合物常用的衍生方法包括硅烷化、烷基化、酰基化等。其中硅烷化试剂主要是三甲基硅衍生试剂,如*N*,*O*-双(三甲基硅基)三氟乙酰胺(BSTFA)-三甲基氯硅烷(TMCS)(99∶1, v/v)混合溶液和MSTFA等;烷基化法的试剂有烯烃、卤代烷烃、硫酸烷酯和醇等;酰基化试剂主要为酰卤、酸酐、酰基咪唑、酰胺及烷基氯甲酸酯等^[12]^。目前使用最为广泛的是BSTFA衍生试剂,反应快、易挥发,BSTFA与TMCS联用可对有位阻的化合物或胺类等较难反应的化合物进行衍生^[13]^。但由于TMCS在衍生反应中用于脱去酸羟基或醇羟基上的质子,促进反应正向发生,而脱去的质子与TMCS上的Cl结合生成HCl,副产物为盐酸,倘若用于在线衍生,大量的HCl势必对色谱柱造成严重损坏。MSTFA与BSTFA衍生效率相似,然而MSTFA及其衍生后的副产物挥发性更强,极易通过溶剂延迟去除,基于该易挥发的特性考虑,本套装置选择MSTFA作为衍生试剂。

衍生试剂阀门3打开后,启动质谱扫描,质谱信号高达近2×10^7^,信号平稳,如图2所示。NIST库检索证实该信号为MSTFA,表明该装置的设计的确使高浓度气态MSTFA随载气(氦气)引入了GC-MS,且浓度恒定。

**图 2:**
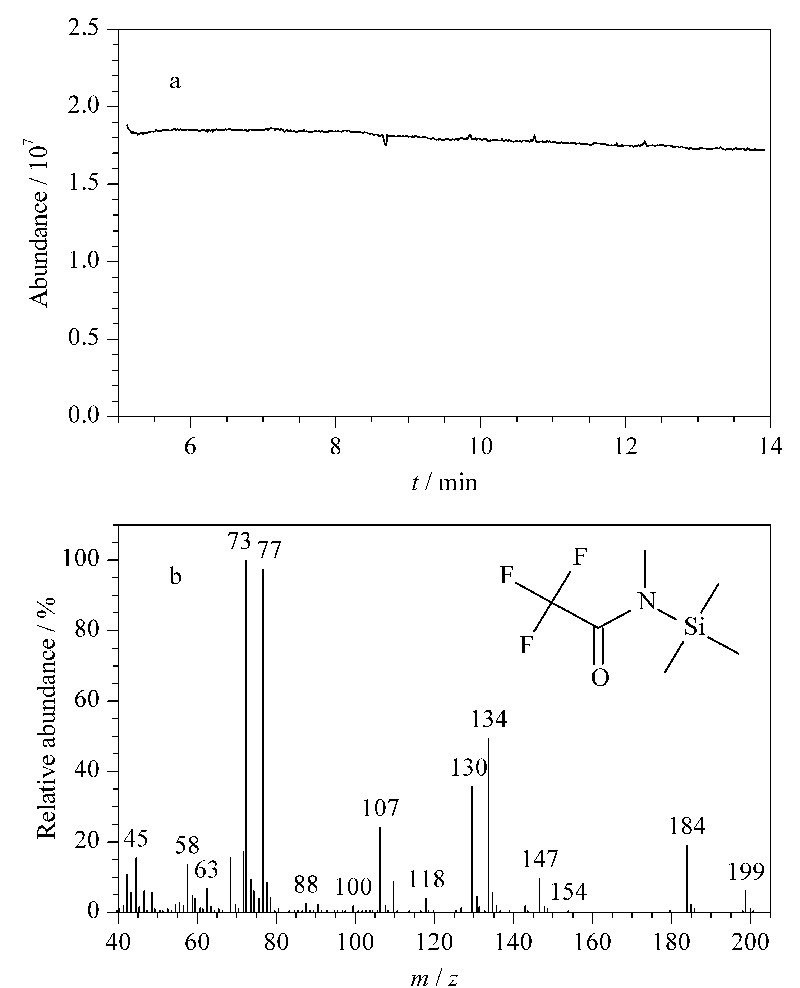
(a)He+MSTFA的GC-MS色谱图和(b)NIST库检索结果

### 2.2 衍生试剂套针设计

设计衍生试剂的导入和引出系统,如图3所示,衍生试剂约1 mL密封在12 mL的玻璃瓶内,瓶口螺帽中心以丁基橡胶垫密封。进样针为套针,针尖上方开侧孔,孔直径为2 mm。不锈钢针头顶部连接三通接头,一根1/32 in的不锈钢管线连入三通侧方,用于引入氦气,该路载气从不锈钢针头侧孔流出;一根内径0.32 μm的石英毛细柱从三通顶部伸入不锈钢针头底部,用于导出气态衍生试剂。从针头侧孔流出的氦气载气对密封的玻璃瓶施压,该压力将瓶内顶空气态衍生试剂从针尖内的毛细柱压入进样口的内衬管,与衬管内滤膜上极性化合物反应,实现衍生。

**图 3:**
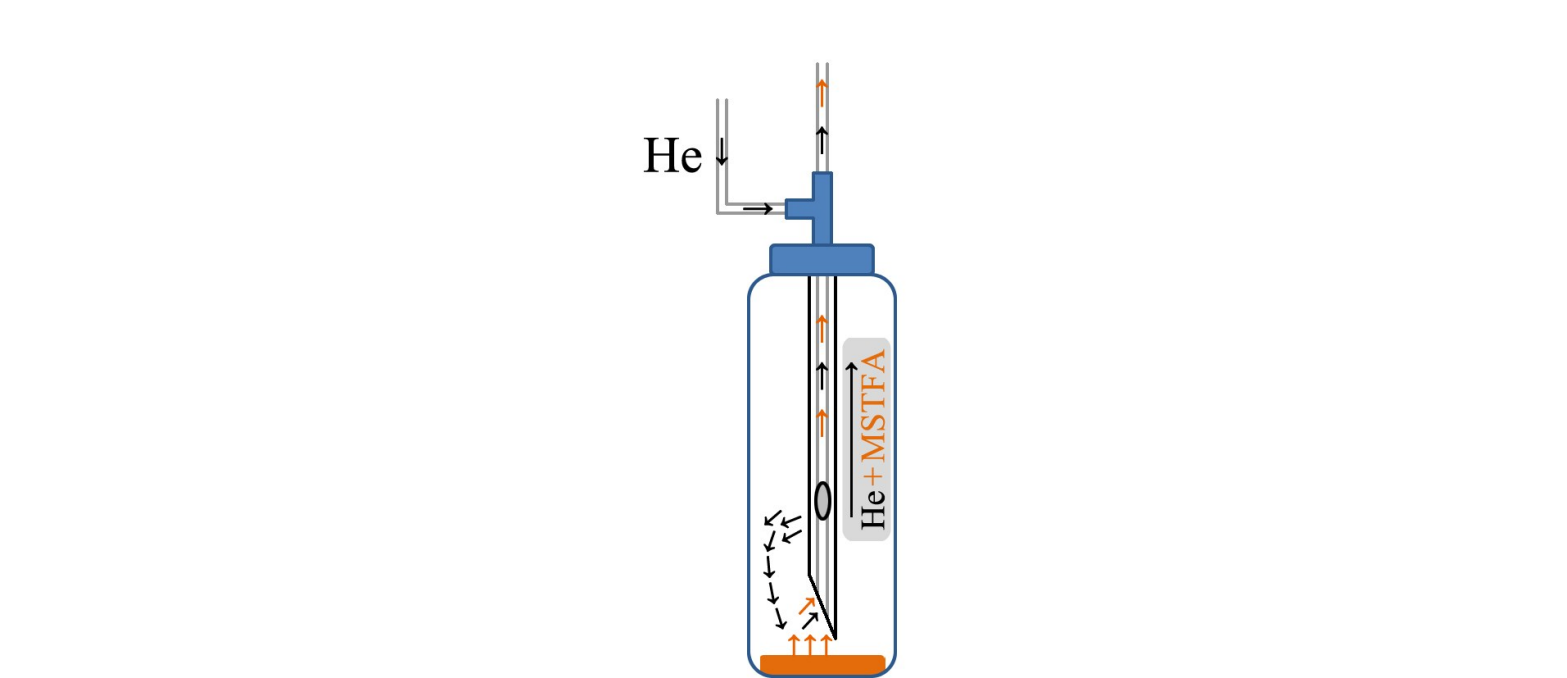
衍生试剂导入针设计图

### 2.3 衍生条件的选择

大气颗粒物中主要的有机酸包括一次排放的碳数小于20的脂肪酸,其中正十六碳酸和十八碳酸与人类活动密切相关,是城市大气颗粒物样品中丰度最高的有机酸组分,占可测出正构烷酸总质量的44%~76%^[14]^,二次排放的有机酸则主要通过苯、甲苯等芳香烃类化合物的芳环断裂降解反应生成二羰基化合物的氧化^[15]^,如丙二酸、丁二酸等二元酸,其余还有芳香酸、酮基酸等。本研究选取了有代表性的4类有机酸:1.一元酸,包括正十六酸和正十八酸;2.二元酸,包括丙二酸、戊二酸、己二酸、辛二酸和壬二酸;3.芳香酸,邻苯二甲酸;4.单蒎烯类氧化产物,包括顺蒎酸和蒎酮酸,共计10种化合物进行条件实验。

2.3.1 衍生温度

进样口温度即衍生反应温度,分别考察290、300、310、320 ℃ 4个温度对各极性化合物衍生反应的影响,如图4所示,结果表明衍生效率随进样口温度升高而提高。但低沸点化合物丙二酸和戊二酸在310 ℃以上,信号值开始下降,高温导致更多样品快速气化后分流出。而己二酸和蒎酸则在310 ℃时发生了信号降低,其余化合物在310 ℃信号基本趋于平稳,综合效率和节能考虑,衍生温度采用310 ℃。

**图 4:**
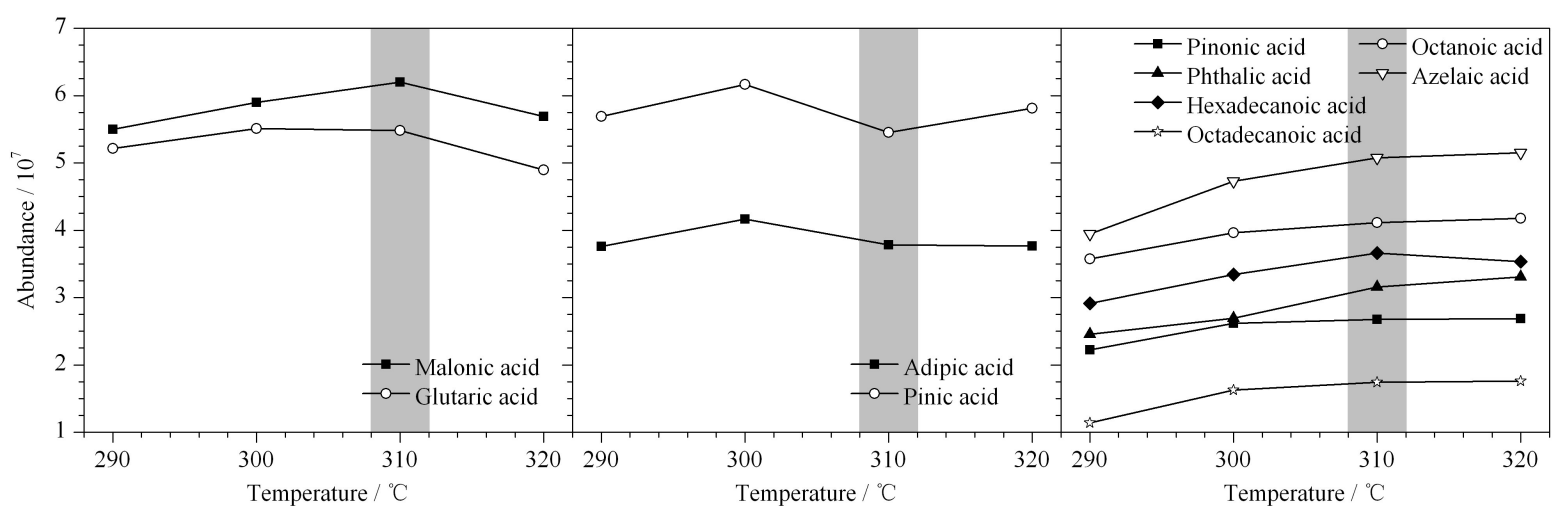
衍生温度对反应效率的影响

2.3.2 衍生时间

考察衍生时间对化合物衍生过程的影响,即进样口温度达到设定的310 ℃高温后,不同保留时间对衍生反应的影响。分别考察0、5、10、15和20 min 5个时间,如图5所示,结果表明:反应初期,衍生时间越长,反应越完全,但10 min以后,丙二酸、戊二酸和邻苯二甲酸信号开始呈现下降趋势,表明样品若已经反应完全并气化,在进样口分流的条件下,保留时间越长,样品将被分流越多,盲目延长衍生时间将会导致样品损失;其余化合物亦在15 min时达到峰值后下降,综合考虑,选择10 min作为衍生时间。

**图 5:**
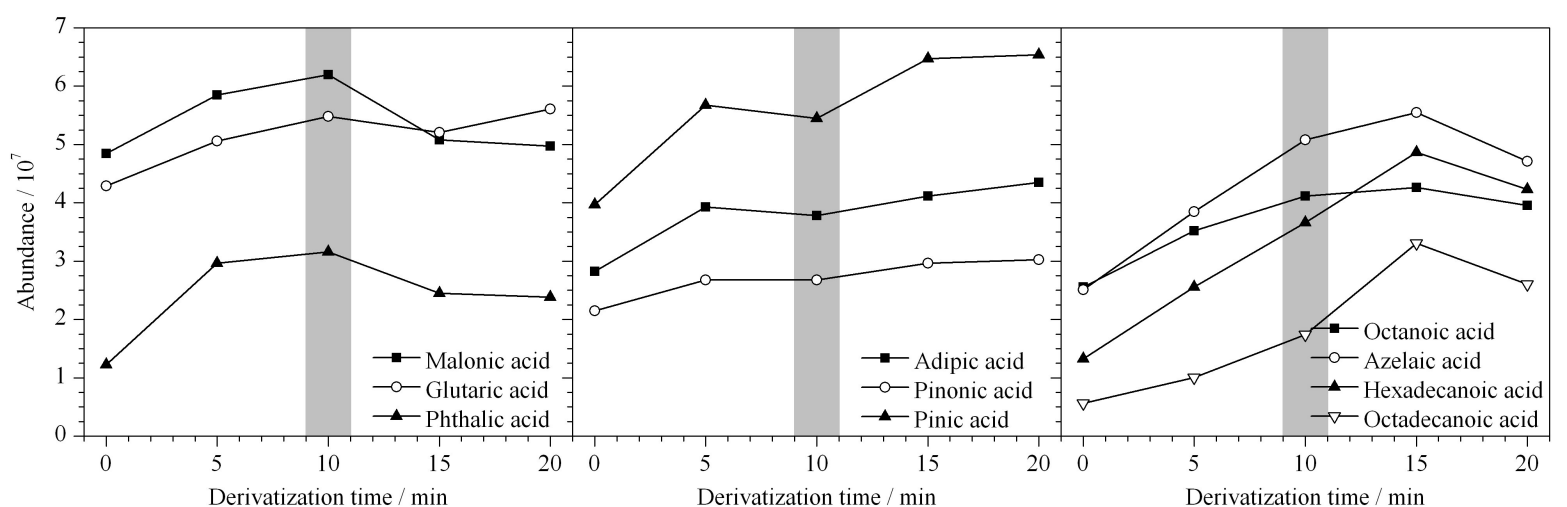
衍生时间对反应效率的影响

### 2.4 方法的线性关系和精密度

配制混合酸标准溶液系列浓度为1、5、10、20、50、100 mg/L,按照1.3节中方法和色谱条件进样分析,并进行线性关系、重复性(日内、日间)和检出限的考察,每个浓度样品分别测定3次,取3次峰面积平均值建立标准曲线,另取50 mg/L的混合标准溶液同日和隔天分别重复进样5次测定峰面积,计算重复性。经过NIST数据库检索,10种有机酸全部完成衍生。标准样品的色谱图见图6,线性关系和精密度结果见表1。

**图 6:**
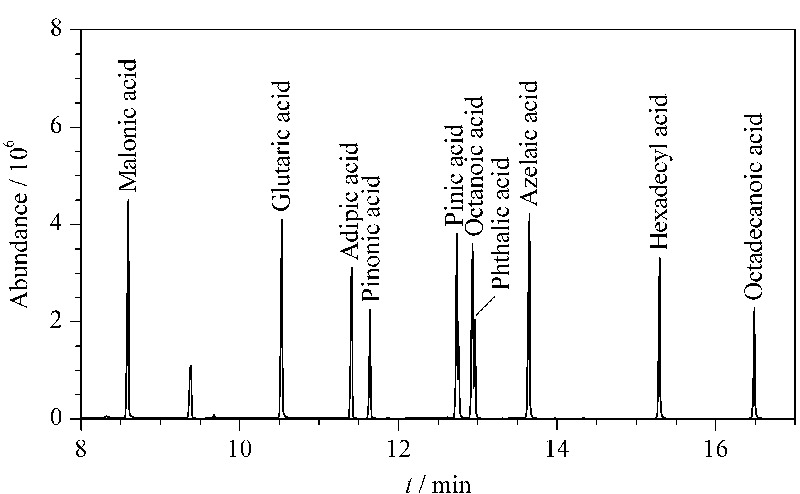
10种有机酸衍生物的总离子流色谱图

**表 1:** 各化合物衍生后主要离子、方法的重复性、线性范围、相关系数和检出限

Compound	Retention time/min	Major ions (m/z)	Derived chemical formula	RSDs/%		Linear range/ (mg/L)	r	LOD/ (mg/L)
				inter-day	intra-day			
Malonic acid	8.27	73, 147, 233	C_9_H_20_O_4_Si_2_	3.51	2.79	2-50	0.993	0.21
Glutaric acid	10.37	73, 147, 261	C_11_H_24_O_4_Si_2_	0.29	0.29	1-100	0.976	0.23
Adipic acid	11.28	111, 147, 175	C_12_H_26_O_4_Si_2_	5.78	0.16	1-100	0.992	0.35
Pinonic acid	11.49	75, 83, 171	C_13_H_25_O_3_Si	1.33	0.44	1-100	0.991	0.17
Pinic acid	12.58	75, 129, 171	C_15_H_32_O_4_Si_2_	7.82	3.85	1-100	0.993	0.05
Octanoic acid	12.78	75, 187, 303	C_14_H_30_O_4_Si_2_	5.39	2.74	1-100	0.996	0.53
Phthalic acid	12.83	73, 147, 295	C_14_H_22_O_4_Si_2_	2.06	3.74	5-100	0.996	0.21
Azelaic acid	13.50	73, 75, 317	C_15_H_32_O_4_Si_2_	5.04	0.86	1-100	0.995	0.07
Hexadecanoic acid	15.13	73, 117, 313	C_19_H_40_O_2_Si	5.09	2.35	2-100	0.989	0.02
Octadecanoic acid	16.33	73, 117, 341	C_21_H_44_O_2_Si	4.43	1.89	2-100	0.973	0.02

### 2.5 实际样品检测

图7为对本研究所上空大气颗粒物进行24 h采集后,滤膜样品在线衍生的全扫描色谱图。由表2可见,通过NIST库进行比对,共计5大类,一共26种极性有机化合物被硅烷化衍生,包括二元酸7种、芳香酸2种、一元酸6种、醇6种、其他酸4种,检出的组分涵盖了大气化学分析常见的大部分目标物,同时NIST库检索表明该方法不仅适用于标样中的有机酸类化合物,同时适用于醇类化合物,包括多元醇类,如葡萄糖醇和山梨糖醇,分子上多达5~6个羟基,存在较大位阻,均全部顺利完成衍生,使得方法适用性进一步扩大。

**图 7:**
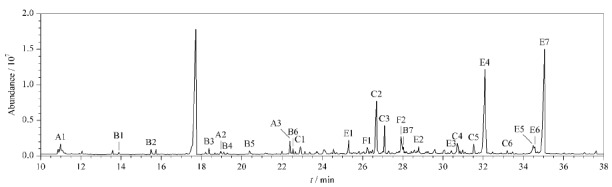
PM2.5样品在线衍生后的色谱图

**表 2:** PM2.5滤膜中检出的有机酸和醇类化合物信息

Type of compounds	Label	Retention time/min	Compound	Major ions (m/z)	Derived chemical formula
Acid	A1	10.99	lactic acid	147, 117, 73, 191	C_9_H_22_O_3_Si_3_
	A2	18.95	glyceric acid	73, 147, 189, 292	C_12_H_30_O_4_Si_3_
	A3	22.40	malic acid	73, 147, 233, 245	C_13_H_30_O_5_Si_3_
	A4	22.94	proline	156, 73, 147, 230	C_11_H_23_NO_3_Si_2_
Diprotic acid	B1	13.90	oxalic acid	73, 147, 66, 190	C_8_H_18_O_4_Si_2_
	B2	15.49	malonic acid	73, 147, 66, 233	C_9_H_20_O_4_Si_2_
	B3	18.37	succinic acid	73, 147, 247, 172	C_10_H_22_O_4_Si_2_
	B4	19.11	fumaric acid	73, 147, 205, 211	C_10_H_20_O_4_Si_2_
	B5	20.39	glutaric acid	73, 147, 261, 158	C_11_H_24_O_4_Si_2_
	B6	22.55	adipic acid	73, 147, 205, 275	C_12_H_26_O_4_Si_2_
	B7	28.03	azelaic acid	73, 129, 201, 317	C_15_H_32_O_4_Si
Aromatic acid	F1	26.26	phthalic acid	73, 75, 147, 295	C_14_H_22_O_4_Si_2_
	F2	27.92	terephthalic acid	295, 221, 103, 73	C_14_H_22_O_4_Si_2_
Alcohol	C1	22.90	erythritol	73, 147, 205, 217	C_16_H_42_O_4_Si_4_
	C2	26.69	levoglucosan	73, 204, 217, 333	C_15_H_34_O_5_Si_3_
	C3	27.09	arabinol	73, 147, 217, 307	C_20_H_52_O_5_Si_5_
	C4	30.75	sorbitol	73, 205, 217, 319	C_24_H_62_O_6_Si_6_
	C5	31.53	glucose	73, 147, 204, 217	C_21_H_52_O_6_Si_5_
	C6	33.18	cyclohexane hexanol	73, 147, 217, 305	C_24_H_60_O_6_Si_6_
Fatty acid	E1	25.31	dodecanoic acid	73, 117, 129, 257	C_15_H_32_O_2_Si
	E2	28.79	tetradecanoic acid	73, 117, 132, 285	C_17_H_36_O_2_Si
	E3	30.41	pentadecanoic acid	117, 299, 73, 129	C_18_H_38_O_2_Si
	E4	32.08	hexadecanoic acid	73, 17, 129, 33	C_19_H_40_O_2_Si
	E5	34.48	linoleic acid	75, 129, 262, 337	C_21_H_40_O_2_Si
	E6	34.57	oleic acid	117, 129, 145, 339	C_21_H_42_O_2_Si
	E7	35.04	octadecanoic acid	73, 117, 132, 341	C_21_H_44_O_2_Si

由图7和表2可见,基于在线衍生装置建立的极性有机物检测方法可直接通过GC-MS对大气颗粒物滤膜样品进行原位分析,同时获得大气颗粒物中多种类有机酸的含量信息。由于不同类型一次排放源产生差异性有机酸,因此可根据有机酸的相对丰度对其来源和贡献进行分析。例如大气颗粒物中含量较高的正十六酸(E4)和正十八酸(E7)与人类活动和生物排放密切相关,在全扫描色谱图(图7)上直观体现,与段凤魁等^[15]^利用传统的溶剂萃取方法报道的结果一致;有机物发生光化学反应形成的二次有机气溶胶是颗粒物中二元有机脂肪酸的主要来源,如甘油酸(A2)和苹果酸(A3)是大气中有机物二次光氧化的主要产物;部分有机酸是分子标记物,是溯源研究的有力工具,如左旋葡聚糖(C2)是含纤维素生物质的热解产物,是生物质燃烧的典型示踪物;葡萄糖、山梨糖醇类多羟基化合物是表层土壤的示踪物;邻苯二甲酸(F1)是塑料制品焚烧的指示物;而不饱和油酸(E6)可在微生物活动及烹饪排放中产生,亚油酸(E5)主要来自烹饪排放等^[16]^。本研究建立的方法可快速定量检测大气颗粒物或细颗粒物中的有机酸,在大气颗粒物污染溯源和大气化学研究中具有广阔的应用前景。

## 3 结论

本工作设计制作了一套用于GC-MS分析极性有机物的在线衍生装置,通过使用气态衍生模式,在进样口衬管对极性化合物实现在线衍生,与传统离线衍生方法相比具有以下优势:1.降低样品需要量。常规溶剂萃取方法分析颗粒物中有机物需采用大流量采样器,需要A4纸大小石英滤膜,而本方法仅需直径2 cm^2^左右的滤膜样品,常规颗粒物采样器(通常为直径47 mm滤膜)即可满足需求;2.衍生反应处于惰性气体氛围,排除了空气中水分对衍生试剂的损耗和衍生产物降解风险,经济高效;3.样品前处理零有机试剂污染,绿色环保;4.装置搭建简单,实验操作简便,易于在不同实验室间推广。目前该装置为手动操作模式,下一步将进行自动化设计,并与热脱附联用,更有利于批量大气颗粒物中极性化合物的快速定量分析。
